# Toxicity and DNA Adduct Formation Reinforce AI-Guided Prediction of Aflatoxin B1 Bioactivation in VERO E6 Cells

**DOI:** 10.3390/toxins18080339

**Published:** 2026-08-04

**Authors:** Bharti Sangwan, Ugochukwu Okoro, Isabella Atteck, Pawel Jaruga, Chinwe Ekenna, Michael Fasullo

**Affiliations:** 1 Department of Nanoscale Science and Engineering, University at Albany, 257 Fuller Road, Albany, NY 12203, USA; 2 Department of Computer Science, University at Albany, 1400 Washington Avenue, Albany, NY 12222, USA; 3 Biomolecular Measurement Division, National Institute of Standards and Technology, 100 Bureau Drive, Gaithersburg, MD 20899, USA

**Keywords:** cytochrome P450, AlphaFold, AutoDoc Vina, aflatoxin B1, DNA adducts

## Abstract

VERO cells, derived from the kidney epithelium of the African green monkey, are widely used in virology, but their ability to metabolize xenobiotics is not fully understood. Since cytochrome P450 (CYP) enzymes participate in xenobiotic metabolism, we investigated which CYP genes are expressed in VERO-E6 cells. Reverse transcription–quantitative polymerase chain reaction (RT-qPCR) showed that VERO-E6 cells express CYP3A4, CYP3A5, and CYP3A7. In contrast, CYP1A1, CYP1A2, CYP1B1, CYP2E1, CYP2D6, and CYP2C9 transcripts were either not detected or at a low detection level. To determine whether the encoded enzymes have the potential to activate aflatoxin B1 (AFB1), we used artificial intelligence (AI)-based structural modeling along with molecular docking. AI modeling suggested that CYP3A enzymes can position AFB1 in an orientation compatible with the formation of the reactive intermediate, and CYP3A4 showed the most favorable predicted interaction (docking score: −16.3 kcal/mol). To demonstrate AFB1 bioactivation, we exposed VERO-E6 cells to 200 nmol/L AFB1. After 10 days, we observed about 40% cell death. Liquid chromatography–tandem mass spectroscopy (LC–MS/MS) analysis confirmed the presence of AFB1-derived DNA adducts, indicating that metabolic activation occurred in these cells. These findings support the presence of CYP-dependent AFB1 bioactivation in VERO-E6 cells. Thus, combining computational and experimental approaches elucidates xenobiotic metabolism in cells where biochemical data are limited.

## 1. Introduction

VERO-E6 cells are derived from the kidney epithelium of the African green monkey (*Chlorocebus sabaeus*) and are widely used as a mammalian cell model in virology (Ammerman et al., 2008) [1], vaccine production (Barrett et al., 2009) [2], and toxicological studies (Costa et al., 2016) [3]. They provide a useful system for studying how cells respond to chemical toxicants because they grow consistently in culture while maintaining intact apoptotic and stress-response pathways (Osada et al., 2014, Romero et al., 2004) [4,5]. As epithelial cells, they serve as a model for the first point of contact for xenobiotics, making them important for studying chemical uptake, cellular injury, and downstream stress responses. This allows investigation of genotoxic mechanisms as well as broader toxicant-induced cellular effects (Gómez-Lechón et al., 2010) [6].

Studies using VERO-E6 cells have already shown how different toxins affect cellular function. For example, microcystin-LR (MCLR) exposure leads to dose- and time-dependent cytotoxicity, mitochondrial dysfunction, oxidative DNA damage, and activation of ERK1/2 signaling pathways (Alverca et al., 2009, Dias et al., 2010, Menezes et al., 2013) [7,8,9]. In a similar way, the mycotoxin ochratoxin A (OTA) has been reported to increase reactive oxygen species (ROS) levels, induce apoptosis, and cause micronucleus formation in VERO cells. These observations suggest that oxidative stress plays a significant role in its toxicity in this system (Costa et al., 2016; Costa et al., 2015) [3,10].

Previous studies have demonstrated AFB1-induced cytotoxicity and genotoxicity in Vero cells under acute exposure conditions using micromolar toxin concentrations (El Golli-Bennour et al., 2010) [11]. However, less is known about AFB1 bioactivation under chronic low-dose exposure conditions or the molecular mechanisms governing CYP-mediated metabolic activation in VERO-E6 cells. Additionally, cellular assays in VERO-E6 cells have been used to detect industrial and environmental pollutants, such as 2,4,6-trichlorophenol, and to assess leachates from drinking water materials, demonstrating that morphological and membrane integrity changes can serve as sensitive indicators of chemical toxicity (Liao et al., 2010) [12]. More recently, VERO-based cytotoxicity datasets have been used to develop naïve Bayesian models for predicting compound toxicity, highlighting their utility in computational toxicology pipelines (Perryman et al., 2018) [13]. These models have been applied to classify structurally diverse small molecules as toxic or non-toxic and to identify chemical features associated with cytotoxic responses. Together, these studies demonstrate that VERO cells are a useful platform for detecting diverse toxicological endpoints.

**Figure 1:**
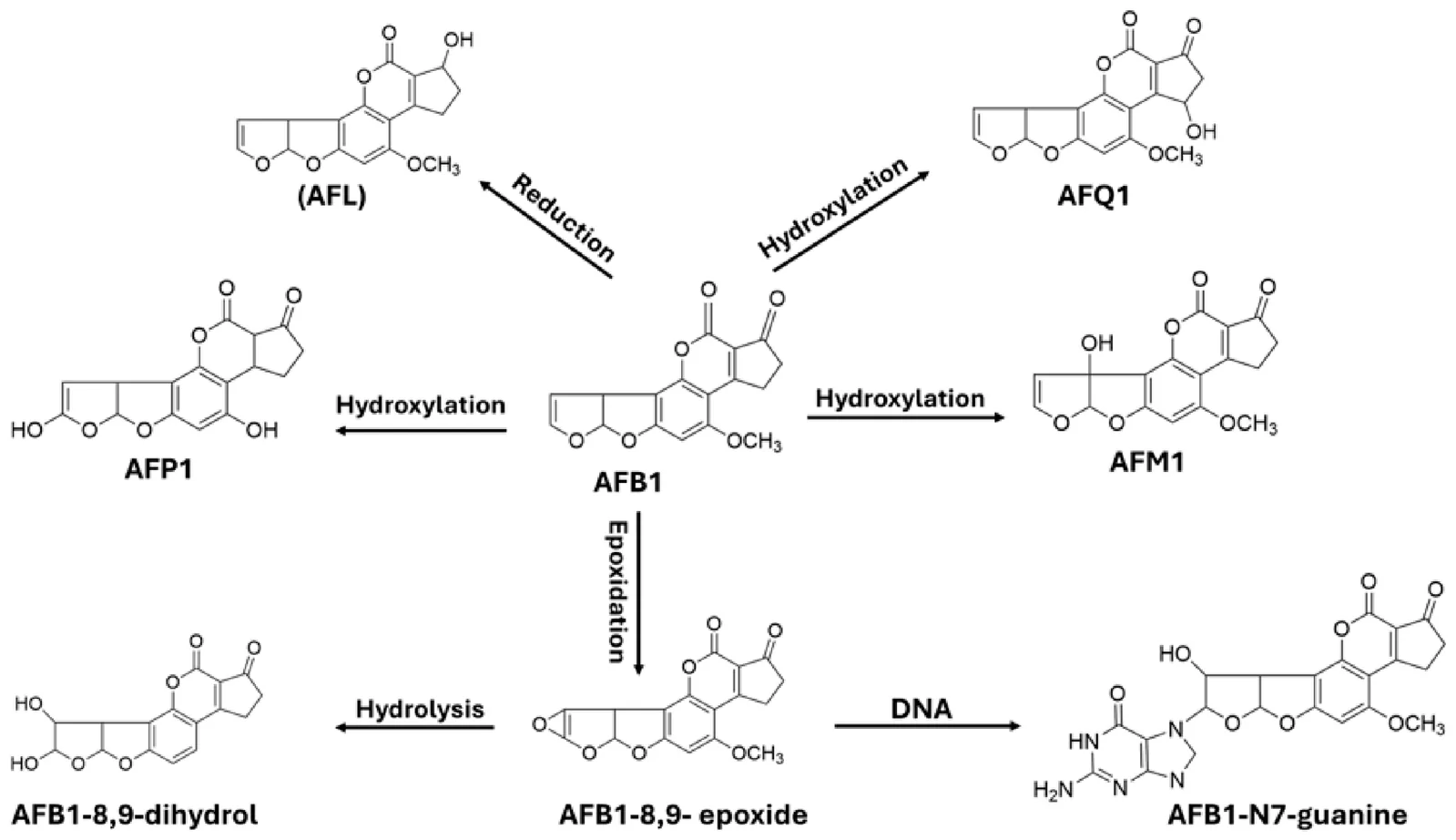
Aflatoxin B1 (AFB1) undergoes enzymatic biotransformation through multiple oxidative and reductive pathways. Hydroxylation reactions produce AFM1, AFQ1, and AFP1, while reduction yields aflatoxicol (AFL). Cytochrome P450-mediated epoxidation at the 8,9-double bond generates the reactive intermediate AFB1-8,9-epoxide, which can be detoxified via hydrolysis to AFB1-8,9-dihydrodiol or react with DNA to form the mutagenic AFB1-N7-guanine adduct (Dohnal et al., 2014) [22].

While the *Chlorocebus sabaeus* genome has been sequenced, revealing orthologs of major *Homo sapiens* (human) cytochrome P450 enzymes involved in xenobiotic metabolism, including CYP1A and CYP3A family members, the metabolic capabilities of VERO cells remain incompletely understood. (Warren et al., 2015) [14]. However, many xenobiotics, including mycotoxins, polyaromatic hydrocarbons, heterocyclic aromatic amines, and small hydrophobic molecules, require metabolic activation by cytochrome P450 (CYP) enzymes to generate reactive intermediates capable of forming DNA adducts and inducing mutagenesis. For example, AFB1 is bioactivated primarily by CYP1A2 and CYP3A family enzymes to form the highly reactive exo-8,9-epoxide (Figure 1), which covalently binds DNA and initiates carcinogenic processes (Kensler et al., 2011, Guengerich et al., 2002) [15,16]. The resulting AFB1-derived DNA adducts, including AFB1-N7-Gua and AFB1-FapyGua, can be sensitively detected and quantified at very low levels by LC-MS/MS, making AFB1 a useful xenobiotic for studying CYP-dependent bioactivation and DNA damage. Recent reviews across multiple animal systems further emphasize that AFB1 toxicity is driven by CYP-mediated bioactivation, oxidative stress, DNA adduct formation, and disruption of cellular homeostasis (Cui et al., 2025) [17].

While hepatic systems are typically used to study such metabolic activation, limited evidence suggests that non-hepatic cell lines, including VERO cells, may express certain CYP enzymes (Gómez-Lechón et al., 2010; Nwabufo et al., 2023) [6,18]. Previous studies have also shown that Vero cells exhibit measurable responses to xenobiotic exposure, including AFB1-induced cytotoxicity and genotoxicity (Golli-Bennour et al., 2010) [11], supporting their utility as a non-hepatic model for studying toxicant metabolism. However, individual cytochrome P450 enzymes from VERO cells have not been purified or systematically characterized at the biochemical level, and their catalytic activity toward well-characterized human CYP substrates remains largely undefined (Guengerich et al., 2008, Zanger & Schwab, 2013) [19,20]. While in vitro studies have demonstrated active cellular metabolism in VERO cells, including glycolytic and mitochondrial pathways (Petiot et al., 2010) [21], direct evidence of functional CYP-mediated xenobiotic metabolism is limited. In particular, protein-level characterization and enzymatic evaluation of key CYP isoforms, especially CYP3A4, have not been well established in VERO-E6 cells. Consequently, the extent to which VERO cells support CYP-dependent metabolic activation of pro-carcinogens remains unclear.

Recent advances in AI-based protein structure prediction have improved structural characterization of enzymes lacking experimentally resolved structures, including CYP enzymes relevant to xenobiotic metabolism. In this study, AlphaFold was used to generate CYP structural models, enabling downstream molecular docking analysis of AFB1 binding and catalytic orientation. In this study, the AI component refers to AlphaFold-based structural modeling of CYP enzymes (Jumper et al., 2021) [23], which was combined with molecular docking to evaluate potential AFB1–CYP interactions. These approaches can help identify candidate CYP isoforms likely to metabolically activate toxicants, predict substrate orientation within catalytic sites, and prioritize enzymes for experimental validation.

However, molecular docking has important limitations. Although docking can predict ligand binding poses and substrate positioning within enzyme active sites, it cannot determine catalytic turnover or confirm metabolic activation. In addition, docking does not account for enzyme expression, protein abundance, cofactor availability, or the cellular environment. Therefore, computational predictions alone are insufficient and require experimental validation.

In this study, we hypothesized that VERO-E6 cells express CYP isoforms capable of metabolically activating AFB1, leading to the formation of reactive intermediates that induce cytotoxicity and DNA adduction. To evaluate this hypothesis, we used an integrated computational and experimental approach to evaluate CYP expression, predict AFB1–CYP interactions, and assess downstream cytotoxicity and DNA adduct formation in VERO-E6 cells.

## 2. Results

### 2.1. Expression of C. sabaeus CYP3A4, CYP3A5, and CYP3A7 RNA Transcripts and CYP3A4 Protein

**Figure 2:**
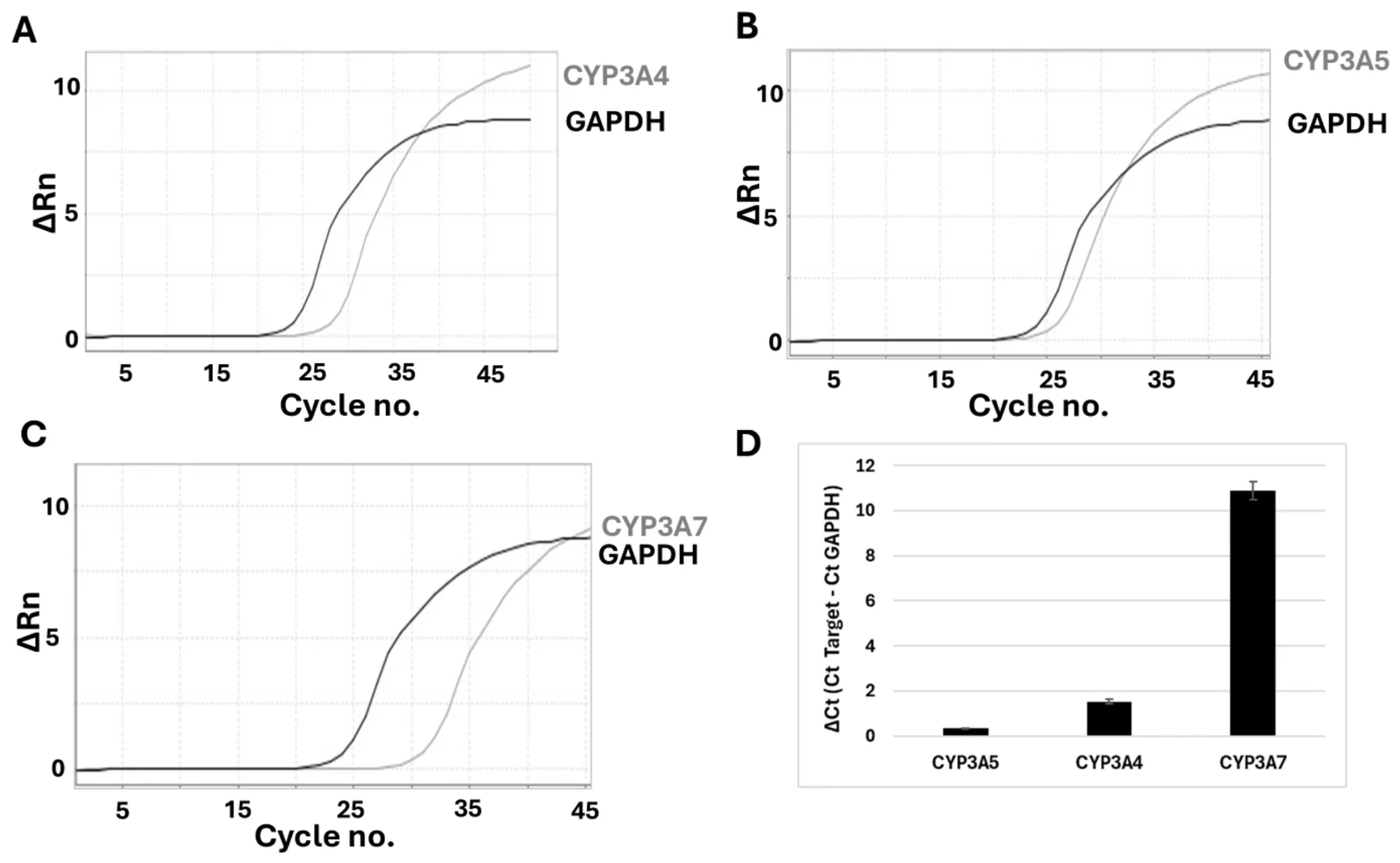
Transcriptional expression of CYP3A4, CYP3A5, and CYP3A7. (**A**–**C**) Representative RT-qPCR amplification curves showing fluorescence signal (ΔRn) versus cycle number for CYP3A4 (**A**), CYP3A5 (**B**), and CYP3A7 (**C**) in VERO-E6 cells. GAPDH was used as the endogenous reference gene. (**D**) Comparison of normalized ΔCt values (Ct_target − Ct_GAPDH) for CYP3A4, CYP3A5, and CYP3A7. Lower ΔCt values indicate higher relative transcript expression. One-way ANOVA demonstrated significant differences among CYP isoforms (*p* < 0.001), and Tukey’s multiple comparison test showed that CYP3A7 differed significantly from both CYP3A5 and CYP3A4 (*p* < 0.001), whereas the difference between CYP3A5 and CYP3A4 was not significant (*p* = 0.082). Data are presented as mean ± SD (*n* = 3).

RT-qPCR analysis was performed to determine CYP gene expression in *C. sabaeus* (VERO-E6) cells (Figure 2). Relative transcript abundance was assessed using ΔCt values (Ct_target − Ct_GAPDH), where lower ΔCt values indicate higher expression relative to GAPDH. Among the CYP isoforms analyzed, CYP3A5 showed the lowest ΔCt value, indicating the highest relative transcript abundance, followed by CYP3A4. To confirm that the CYP3A4 mRNA was also translated, we performed a Western blot (Supplementary Figure S1). In contrast, CYP3A7 exhibited a substantially higher ΔCt value, consistent with markedly lower transcript abundance. One-way ANOVA of ΔCt values demonstrated significant differences among CYP isoforms (*p* < 0.001). Post hoc Tukey’s multiple comparison analysis showed that CYP3A7 expression differed significantly from both CYP3A5 and CYP3A4 (*p* < 0.001), whereas the difference between CYP3A5 and CYP3A4 was not statistically significant (*p* = 0.082). CYP1A2, CYP1B1, and CYP2E1 showed very high Ct values (≈39–42), indicating extremely low transcript abundance near the detection limit. CYP1A1, CYP2D6, and CYP2C9 were not detected under these conditions. Overall, these results indicate that VERO-E6 cells express detectable CYP3A-family transcripts, with CYP3A5 and CYP3A4 showing the highest relative expression in this dataset.

### 2.2. Comparison of the H. sapiens and C. sabaeus CYP3A Amino Acid Sequences

We observed close similarities between the amino acid sequences of the CYP3A enzymes from *C. sabaeus* (VERO E6) and those of human versions (Supplementary Figure S2), which were all approximately 500 amino acids in length. Among the individual isoforms, CYP3A4 showed the highest similarity, with about 93% identity and 97% similarity between *C. sabaeus* and human sequences. CYP3A5 and CYP3A7 isoforms were also quite similar, with roughly 91% identity and 95% similarity. Aligned sequences exhibited at most one gap, indicating few insertions or deletions. Some similarity is due to conservative substitutions, where the amino acid changes do not affect charge or hydrophobicity. The catalytic regions were the most conserved, including key motifs and the substrate recognition sites (SRS1–SRS6), which are important for enzyme function. For example, the heme-binding motif (FGxGPRNCIG), which contains the cysteine that coordinates the heme iron, was conserved in all three CYP3A proteins. In *C. sabaeus*, the “x” position is threonine (T), while in humans it is serine (S), which is still a conservative change. Other motifs, such as PERF and EXXR, were also present in both species, suggesting that the catalytic core is maintained. On the other hand, most of the differences were seen in the N-terminal region and in some surface-exposed loops. The apparent flexibility of the surface-exposed loops suggests that these changes have minimal effects. The sequences are still quite similar overall, and the key catalytic features are present in both species. Based on this, CYP3A enzymes in *C. sabaeus* and humans likely share functional similarities.

### 2.3. Conservation of the H. sapiens and C. sabaeus Three-Dimensional CYP Structures

**Figure 3:**
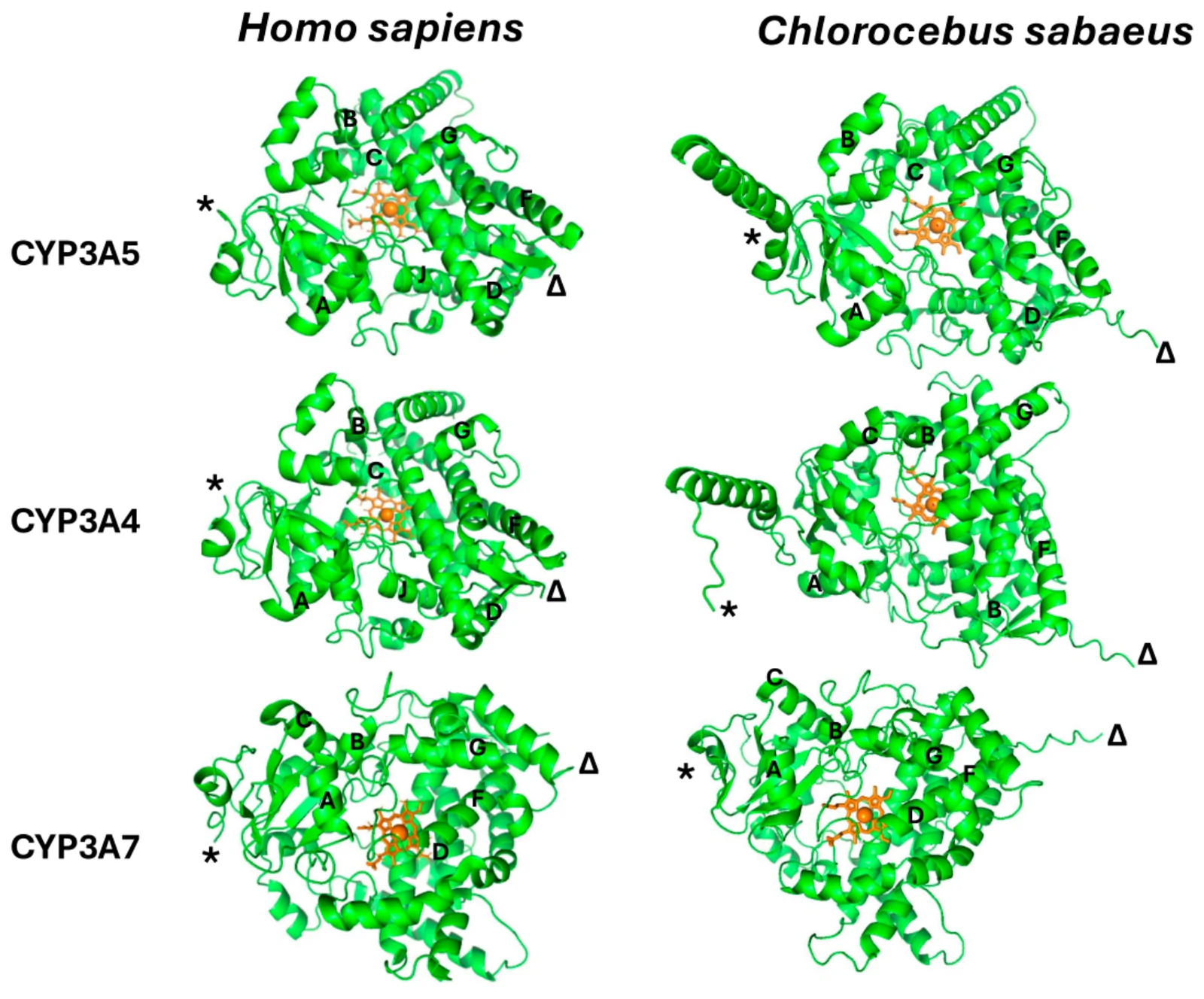
Structural comparison of *H. sapiens* and *C. sabaeus* CYP3A isoforms. Experimentally determined human CYP3A5 (PDB: 5VEU), CYP3A4 (PDB: 9BV6), and CYP3A7 (PDB: 8GK3) structures (**left**) were compared with AlphaFold-predicted structures of the corresponding C. sabaeus (VERO E6) CYP proteins (**right**). Structures are shown in ribbon representation with the heme prosthetic group centered within the catalytic pocket. The overall cytochrome P450 fold is conserved across species and is characterized by a predominantly α-helical architecture (green) surrounding the heme active site (orange). N-terminal regions are indicated by asterisks (*), and C-terminal regions are indicated by delta symbols (∆), highlighting differences in terminal orientation and surface loop extensions between species. Major α-helices (A, B, C, D, F, and G) are labeled to illustrate conserved structural elements involved in shaping the catalytic cavity and substrate access channels. The J helix of CYP3A4 and CYP3A5 present in *H. sapiens* is not present in the *C. sabaeus* CYP3A4 and CYP3A5.

To determine whether *C. sabaeus* CYP enzymes have the structural features needed for catalysis, we compared AlphaFold-predicted structures with experimentally determined human CYP structures, including CYP3A5 (PDB: 5VEU) (Hsu et al., 2018) [24], CYP3A4 (PDB: 9BV6) (Wang et al., 2025) [25] and CYP3A7 (PDB: 8GK3) (Liu et al., 2023) [26] (Figure 3). All *C. sabaeus* CYP3A isoforms exhibited the typical cytochrome P450 fold, including the heme-binding catalytic core. Overall, the fold and the position of the heme were similar to those seen in human enzymes, suggesting that the basic catalytic structure is preserved.

Notable differences were observed in substrate access regions, particularly the B–C and F–G loops, which appeared slightly more flexible and extended in *C. sabaeus* enzymes. Since these loops are known to control substrate entry into the active site, this added flexibility may facilitate ligand entry and accommodation without affecting the core catalytic structure. A comparison of human and *C. sabaeus* CYP3A isoforms is summarized in Table 1, which highlights a conserved catalytic core and minor differences in loop flexibility and active-site openness. Another difference was observed in the N-terminal region between human and *C. sabaeus* CYP3A isoforms. This region stable association of the enzymes associated with the endoplasmic reticulum membrane. In many human CYP crystal structures, this part is often missing or only partially resolved because it is flexible and difficult to crystallize. In contrast, the AlphaFold models include the full-length protein, so the N-terminal region looks more extended and flexible. Because of this, its orientation appears different from that seen in human structures. That said, this region is mainly involved in anchoring the protein to the membrane and is not close to the catalytic site. So, any differences here are unlikely to have a major effect on the heme-binding pocket or enzyme activity. From this, it seems that *C. sabaeus* CYP3A enzymes still retain the key structural features needed for substrate binding and catalysis.

### 2.4. Multiple Docking Orientations Reveal Accessible Binding Landscapes

**Figure 4:**
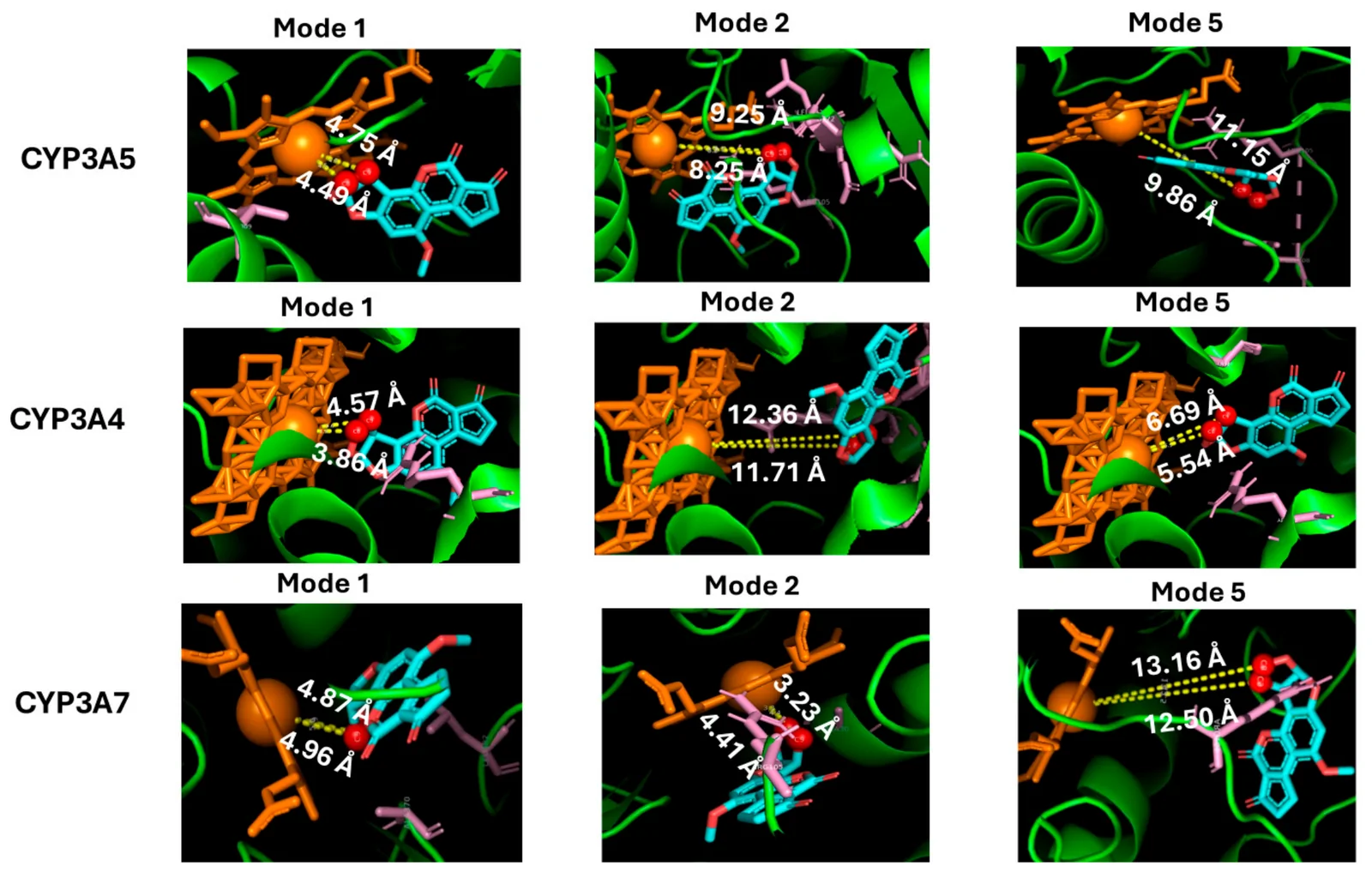
Representative zoomed-in views of the active sites showing energetically favorable docking poses of aflatoxin B1 (AFB1) within CYP3A5 (**top** row), CYP3A4 (**middle** row), and CYP3A7 (**bottom** row). For each enzyme, selected binding modes (Mode 1, Mode 2, and Mode 5) illustrate alternative ligand orientations relative to the heme prosthetic group. The active site is surrounded by α-helices, shown in green. AFB1 is shown in cyan (blue), the heme prosthetic group is shown in orange with the heme iron represented as a large orange sphere. Interacting amino acids are shown in purple. Yellow dashed lines indicate the measured distances (Å) between the heme iron and the reactive C8 and C9 atoms of AFB1, which are shown as red spheres in the enlarged views of the catalytic pocket. The oxygen atoms of AFB1 are shown in red.

We used molecular docking to look at how AFB1 might interact with CYP enzymes. In the simulations, AFB1 did not stay in a single position within the CYP3A active site (Figure 4). Instead, it adopted several different binding orientations. We observed differences in how deeply the ligand positioned in the pocket, how it was oriented with respect to the heme, and where it was oriented along the access channel. Overall, this suggests that the CYP binding pocket is quite flexible. Across docking runs, AFB1 was observed in both the central catalytic region and the nearby access channel. In some cases, such as CYP3A5 Mode 1 and CYP3A4 Mode 1 (Figure 4), the difuran moiety of AFB1 was oriented toward the heme group. In other poses, including CYP3A4 Mode 2 and CYP3A7 Mode 3 (Figure 4), the coumarin ring was directed toward the catalytic center. These differences indicate that AFB1 can reposition within the active site, allowing distinct parts of the molecule to approach the heme iron during docking.

Analysis of binding energies indicated that AFB1 interacts well with all CYP3A isoforms (Table 2). Among the isoforms, CYP3A4 generally had the lowest binding energies, suggesting that it accommodates AFB1 more tightly. In comparison, CYP3A5 and CYP3A7 also had favorable interactions, but to a lesser extent. These differences are probably due to minor changes in active-site shape, residue composition, and the flexibility of the access channel in each isoform.

A more detailed look at individual docking modes is summarized in Table 3. In the productive poses, AFB1 stabilization within the active site involved several residues located along the catalytic cavity and access channel. For CYP3A4, we kept seeing the same set of residues come up as Phe108, Phe213, Phe215, Phe241, and Phe304. These are mostly aromatic and hydrophobic, and they seem to form a kind of pocket that holds the planar rings of AFB1 in place, probably through hydrophobic and π–π interactions. Leu482 is also part of this region and adds to the hydrophobic surface. Residues like Arg105 and Arg212 are nearby as well and seem to help with how the ligand sits in the pocket. CYP3A5 did not look the same. Thr309 is close to the catalytic cavity and might be involved in positioning the ligand, possibly through polar interactions or hydrogen bonding. Arg105 and Phe213 are still there, but the interactions here seem less dominated by hydrophobic contacts and more by polar ones. In CYP3A7, the favorable poses were mainly associated with arginine residues, such as Arg105 and Arg372, as well as hydrophobic residues, such as Ala305, Ala370, and Leu482. In this case, AFB1 seems to be stabilized more by a mix of electrostatic interactions and hydrophobic contacts than by the strong aromatic interactions we see in CYP3A4. The comparison of the three most catalytically relevant AFB1 docking poses for *H. sapiens* and *C. sabaeus* CYP3A isoforms are compared in (Supplementary Table S1).

Importantly, a high binding energy alone does not necessarily indicate that catalysis will occur. Several poses with favorable docking scores placed AFB1 too far from the heme center (Table 3), making them unlikely to support epoxidation. For catalytic activation, the reactive C8–C9 double bond of the difuran ring needs to be positioned close to the heme iron–oxo species. Only those orientations that bring this region near the heme are likely to lead to the formation of the AFB1-8,9-exo-epoxide, which is responsible for DNA adduction. The differences in residues involved in stabilizing these poses likely reflect small variations in active-site structure and flexibility among the CYP3A isoforms. Although the overall fold and heme-binding architecture are conserved, even minor differences in side-chain composition can influence how the ligand is positioned (Ekroos & Sjögren, 2006) [27]. As a result, each CYP isoform appears to use a slightly different combination of aromatic, hydrophobic, and polar residues to orient AFB1 for catalysis. The fact that we observe multiple favorable orientations aligns with what is already known about CYP enzymes, which are quite flexible and allow substrates to move within the active site before settling into a productive position. Overall, this suggests that CYP3A enzymes can bind AFB1 in multiple ways. This flexibility likely helps the ligand adjust its position until the reactive difuran region is aligned with the heme catalytic center.

### 2.5. Catalytically Favorable Binding Orientations Support Epoxidation Potential

**Figure 5:**
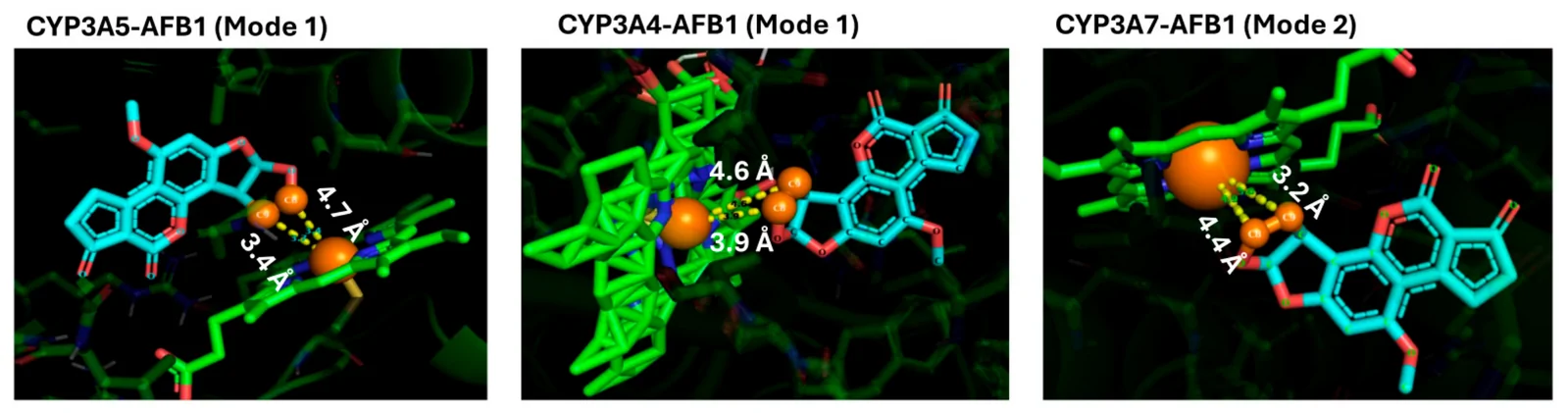
Representative epoxidation-competent docking poses of AFB1 within the active sites of C. sabaeus CYP3A5, CYP3A4, and CYP3A7. AFB1 is depicted in cyan, the heme group in green, the nitrogens of the porphyrin ring are blue, oxygen atoms are red, and iron is an orange sphere. Distances between the heme iron and the C8 and C9 atoms of AFB1 are indicated. In CYP3A5, the C8–C9 double bond is positioned at 3.4 Å and 4.7 Å from the heme iron, while in CYP3A4, corresponding distances of approximately 3.9 Å and 4.6 Å are observed, and in CYP3A7, the C8-C9 double bond is positioned at 3.2 Å and 4.4 Å. These distances allow for catalytic transfer of oxygen to the C8–C9 double bond, indicating epoxidation-competent geometry.

Among the docking results, we selected representative poses with both low binding energies and favorable catalytic geometries for closer analysis (Figure 5). For AFB1 to be metabolically activated, the reactive C8–C9 double bond needs to be positioned close to the heme iron so that oxygen transfer can occur and the AFB1-8,9-epoxide can form. Based on this, we examined the docking poses by considering the ligand’s orientation, its distance from the heme, and its fit within the active site. In the selected poses, AFB1 was generally positioned with its difuran ring facing the heme, bringing the C8–C9 bond close enough to potentially support catalysis. Among the CYP isoforms, CYP3A4 placed AFB1 closest to the heme iron, which likely makes it the most favorable for epoxidation. CYP3A5 and CYP3A7 exhibited slightly larger Fe–C8/C9 distances, but the ligand was still positioned in a way that could support catalytic activation. This suggests that all three enzymes may be capable of forming epoxide.

The active-site views in Figure 5 indicate that these productive orientations are stabilized by interactions between AFB1 and residues lining the catalytic cavity. Aromatic residues appear to form hydrophobic and π–π interactions with the planar aflatoxin structure, helping to hold the ligand in place above the heme. We also noticed that nearby polar and charged residues seem to affect how the ligand sits in the pocket and limit how much it moves. Because of that, the reactive double bond tends to stay pointed toward the catalytic center.

Taken together, this kind of positioning looks suitable for oxygen insertion. The details of the different modes, including Fe–C8/C9 distances and interacting residues, are listed in Table 3. From this, it is clear that catalytic potential is not determined by binding energy alone. For example, some poses with good docking scores, such as CYP3A4 Mode 2 and CYP3A5 Mode (Table 3), placed the ligand away from the heme and are therefore unlikely to support epoxidation at the C8–C9 position. These poses may reflect alternative ways the ligand can bind within the active site, but the orientation does not appear suitable for oxidation at the main reactive site. In contrast, the poses shown in Figure 5 not only have good binding energies but also place the ligand closer to the heme with supportive interactions, keeping the C8–C9 bond near the catalytic center. These poses are therefore more likely to reflect orientations that can support catalysis. The way CYP3A enzymes position AFB1 here also fits with what is already known about their flexibility and ability to work with different substrates. The minor differences in Fe–C8/C9 distances between the isoforms likely come from differences in active-site shape or how flexible the surrounding loops are, which may influence how efficiently the reaction proceeds. Overall, these results suggest that *C. sabaeus* CYP enzymes are structurally capable of positioning AFB1 in orientations that support formation of the reactive epoxide intermediate responsible for DNA adduct formation.

### 2.6. DNA Adduction Confirms AFB1 Bioactivation

**Figure 6:**
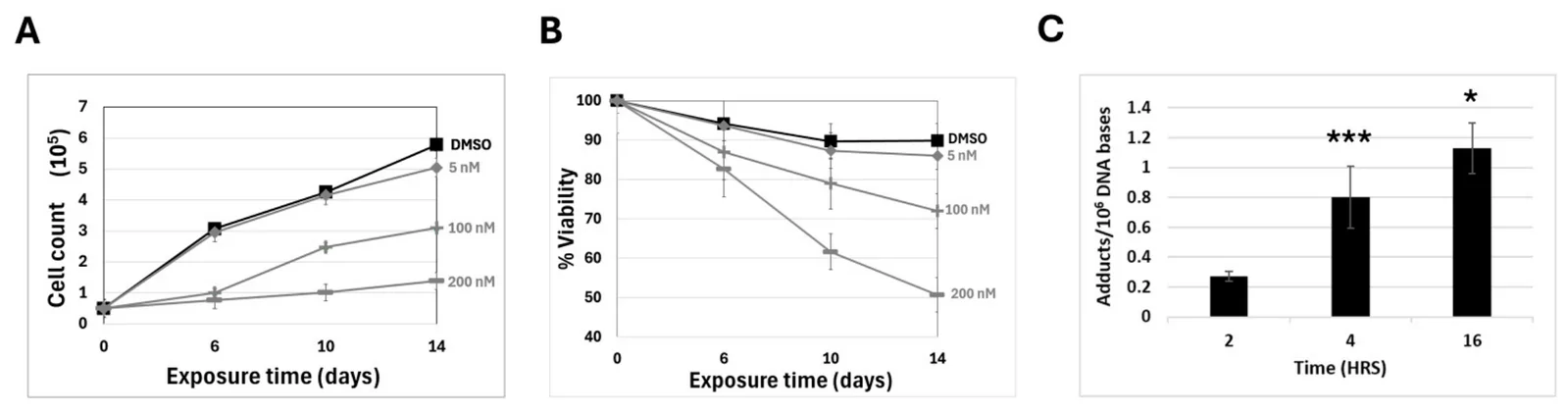
VERO cell viability and proliferation after AFB1 exposure. (**A**) VERO cells were exposed to increasing concentrations of AFB1 (5 nmol/L to 200 nmol/L), and cell proliferation was monitored over 14 d. (**B**) Percent cell viability was assessed over the same time period. DMSO-treated cells served as controls. AFB1 caused a concentration- and time-dependent reduction in cell number and viability, with 200 nmol/L producing the greatest loss of growth and survival by day 14. Data represent mean ± SD. (**C**) Total DNA adduct levels following exposure to 200 nmol/L AFB1 increased over time, indicating progressive DNA damage. Significance was determined using one-way ANOVA followed by Dunnett’s test with 2 h as the reference; asterisks indicate (***) *p* < 0.001 and (*) *p* < 0.05, which denote significant increases relative to the 2 h time point.

The most direct evidence of metabolic activation in VERO E6 cells is the formation of AFB1-derived DNA adducts. We quantified AFB1-derived DNA adducts following short-term exposure. Cells were treated with 200 nmol/L AFB1 for 2 h, 4 h, and 16 h, and total DNA adducts, including the AFB1 N^7^Gua and AFB1 Fapy adducts, were quantified (Figure 6C). After 4 h of exposure to 200 nmol/L AFB1, total DNA adduct levels increased approximately fourfold relative to 2 h treatment. The maximum adduct burden reached approximately 1.2 adducts per 1000 kb of DNA at 16 h following a single exposure. Statistical analysis (one-way ANOVA followed by Dunnett’s test, *p* < 0.001 at 4 h and *p* < 0.05 at 16 h) confirmed significant increases in adduct formation relative to the 2 h reference time point. These results support that AFB1 is rapidly bioactivated in VERO-E6 cells, resulting in the formation of standard genotoxic DNA lesions.

### 2.7. AFB1 Induces Time- and Dose-Dependent Cytotoxicity

Consistent with the DNA adduct data, AFB1 exposure caused pronounced cytotoxicity in VERO-E6 cells. Dose- and time-dependent reductions in viability were observed, with approximately 50% lethality by 14 d after prolonged exposure to 200 nmol/L AFB1 (Figure 6A,B). The data indicated that AFB1 exposure not only killed cells but also reduced cell number. This delayed cytotoxic response is similar to that reported in hepatocyte-based models, in which toxicity is mainly linked to the accumulation of reactive metabolites rather than to direct chemical damage (Kensler et al., 2011) [15]. Two-way ANOVA of cell number data demonstrated significant main effects of exposure time (*p* = 3.0 × 10^−6^) and AFB1 concentration (*p* = 2.08 × 10^−4^), whereas the interaction between time and dose was not statistically significant (*p* = 0.108). Post hoc Dunnett’s multiple comparison test showed that 200 nmol/L AFB1 significantly reduced cell numbers at all examined points relative to DMSO controls, including day 6 (*p* = 0.0005), day 10 (*p* = 0.0021), and day 14 (*p* = 0.00003). At 100 nmol/L, significant reductions in cell numbers were observed on day 10 (*p* = 0.0008) and day 14 (*p* = 0.0004), whereas no significant effect was detected on day 6. In contrast, 5 nmol/L caused only minor and inconsistent changes that were not significant after correction for multiple comparisons.

A similar pattern was observed for percent viability. Two-way ANOVA demonstrated highly significant effects of exposure time (*p* = 1.10 × 10^−31^), AFB1 concentration (*p* = 8.57 × 10^−28^), and a significant interaction between time and dose (*p* = 2.62 × 10^−21^), indicating that the cytotoxic effect of AFB1 increased with both concentration and duration of exposure. Post hoc Dunnett’s comparisons showed that 200 nmol/L AFB1 significantly reduced viability at all times compared with DMSO controls, including day 6 (*p* = 0.002), day 10 (*p* = 0.007), and day 14 (*p* = 0.0012). At 100 nmol/L, significant decreases in viability were observed beginning at day 6 (*p* = 0.048) and became more pronounced at day 10 (*p* = 0.039) and day 14 (*p* = 0.00004). At 5 nmol/L, no significant reduction in viability was observed at day 6 or day 10, whereas a modest decrease was detected at day 14 (*p* = 0.015). Overall, these results indicate that AFB1 cytotoxicity in VERO-E6 cells depends strongly on both dose and exposure duration. Notably, reductions in cell number were observed before major decreases in viability, suggesting that AFB1 initially suppresses cell proliferation prior to inducing more pronounced cell death during prolonged exposure.

## 3. Discussion

The present study integrates molecular, structural, computational, and biochemical approaches to evaluate the metabolic competence of VERO-E6 cells for AFB1 bioactivation. The RT-qPCR and computational analyses provide predictive evidence that VERO-E6 cells express CYP isoforms with structural features compatible with AFB1 binding and possible catalytic activation. Specifically, CYP3A4, CYP3A5, and CYP3A7 transcripts were detected, and AlphaFold-based structural models indicated that these enzymes retain the typical cytochrome P450 fold. Molecular docking further suggested that AFB1 can adopt orientations within CYP3A active sites that place the reactive C8–C9 bond near the heme center, particularly in CYP3A4. In contrast, the experimental evidence comes from the observed AFB1-induced cytotoxicity and LC-MS/MS detection of AFB1-derived DNA adducts in treated VERO-E6 cells. These findings support that AFB1 bioactivation occurred in this cell system. The different time scales of DNA adduct and cytotoxicity analyses reflect distinct stages of AFB1-induced toxicity. DNA adduct formation represents an early genotoxic event following AFB1 bioactivation, whereas reduced cell proliferation and viability reflect downstream consequences of accumulated cellular damage during prolonged exposure. Early sampling was also important to detect AFB1-N7-Gua, an unstable adduct that can rapidly undergo depurination, repair, or conversion to more stable Fapy adducts. However, the current data does not directly establish which CYP isoform is responsible for adduct formation, and CYP3A involvement should therefore be interpreted as suggested by transcript expression and docking rather than definitively demonstrated. Our findings are broadly consistent with established hepatic models of AFB1 bioactivation, in which CYP1A2 and CYP3A4 are recognized as major enzymes responsible for the conversion of AFB1 to the reactive exo-8,9-epoxide (Guengerich et al., 2002; Gallagher et al., 1996; Cui et al., 2025) [16,17,28]. In hepatic systems, CYP1A2 often contributes substantially at lower substrate concentrations, whereas CYP3A4 becomes increasingly important at higher exposure levels. In contrast, VERO-E6 cells showed detectable expressions of CYP3A4, CYP3A5, and CYP3A7, but minimal CYP1A2 expression, suggesting that AFB1 bioactivation in this model may rely predominantly on CYP3A-family enzymes. Although the metabolic capacity of VERO-E6 cells is expected to be lower than that of hepatic systems, the detection of AFB1-derived DNA adducts demonstrates that these cells retain sufficient bioactivation capability to support mechanistic toxicology studies. Together, these results support the value of combining computational prediction with experimental validation to investigate xenobiotic bioactivation in cell systems where direct enzymatic characterization is limited.

Here, we used AI-based structural modeling and molecular docking to explore CYP-mediated metabolic activation, especially since detailed biochemical data for this system are limited. In VERO-E6 cells, although CYP gene expression can be detected, the lack of purified enzymes and limited functional data make it difficult to directly study enzyme–substrate interactions using conventional methods. Using AI-predicted protein structures, we were able to model the three-dimensional organization of VERO CYP enzymes and examine the active site in more detail. With molecular docking, we looked more closely at how AFB1 might interact with these enzymes—specifically, how it sits in the binding pocket, which residues are nearby, and how close it gets to the heme iron. For example, we determined whether the reactive C8–C9 double bond of AFB1 could be positioned within the substrate binding site in a manner that would preferentially favor epoxide formation. At the same time, there are some limitations with this approach. In most docking methods, the protein is treated as rigid, which does not reflect all dynamic structures (Lee et al., 2025) [29]. We also noticed that scoring functions can sometimes rank poses as favorable just based on energy, even if those poses are not likely to be relevant for catalysis (Desta et al., 2020) [30]. Therefore, binding affinity alone is insufficient to predict metabolic activation. In CYP enzymes, productive oxidation depends not only on ligand binding but also on proper substrate orientation relative to the heme center and protein conformational flexibility. As a result, some predicted strong AFB1 interactions may represent false-positive binding poses that do not lead to efficient epoxide formation.

Another limitation is that these computational methods do not give any information on how the biological context of the enzyme can affect activity (Zanger & Schwab, 2013) [20]. In cells, CYP activity is influenced by factors that are not accounted for by the folded structure predicted by the primary sequence, including translational modifications, orientation of the enzyme within the endoplasmic reticulum membrane, its interaction with cytochrome P450 oxidoreductase (POR), and the local membrane environment. Among these factors, POR plays a critical role by transferring electrons from NADPH to the CYP heme center, enabling oxygen activation and catalytic substrate oxidation. Without efficient electron transfer from POR, substrate binding alone may not result in productive metabolism. Therefore, variation in POR expression or CYP–POR interactions may influence AFB1 bioactivation efficiency in VERO-E6 cells.

From the structural and docking analysis, CYP3A4 is best suited to bind AFB1 in the most favorable position for epoxidation. However, additional functional studies are needed to determine which CYP isoform is mainly responsible for this activity in VERO-E6 cells.

When we compared the *H. sapiens* and *C. sabaeus* CYP3A enzymes, we observed high sequence and structural similarity, particularly in the catalytic core and heme-binding region. We also saw that those important motifs for enzyme activity, such as the conserved cysteine for heme binding and the substrate recognition sites, were present in both species. This points to the enzymes working in an equivalent way. We also noticed some differences in the N-terminal region, which engages in membrane anchoring and may influence how the enzyme is positioned in the endoplasmic reticulum (Denisov et al., 2005) [31]. We also noticed variations in the B–C and F–G loop regions. These regions are quite flexible and seem to be involved in how substrates get into the active site. Because of that, any differences here are more likely to affect how the substrate enters rather than the catalytic step itself (Yan & Hirao, 2026, Li et al., 2022, Urban et al., 2018) [32,33,34].

The CYP3A family plays a major role in AFB1 metabolism, with CYP3A4 recognized as one of the primary enzymes involved in bioactivation of AFB1 to the reactive exo-8,9-epoxide (Zanger & Schwab, 2013) [20]. Based on the structural similarity observed in this study, VERO CYP3A enzymes may interact with AFB1 in a manner comparable to human CYP3A enzymes. The favorable docking orientation of AFB1 within the CYP3A4 active site supports its potential role in facilitating epoxide formation, which is consistent with the experimental detection of AFB1-derived DNA adducts. These findings suggest that AI-based structural modeling and docking can provide useful mechanistic insight into AFB1 bioactivation and toxicological responses in VERO-E6 cells (Jumper et al., 2021, Stokes et al., 2020) [23,35].

Beyond mechanistic toxicology, these findings also have relevance for aflatoxin monitoring and food safety assessment. While analytical methods can detect AFB1 contamination at extremely low levels, toxicological risk depends not only on exposure but also on metabolic activation and detoxification. Biomarkers such as AFB1-derived DNA adducts therefore provide an important link between exposure and biological effect. These mechanistic findings also have practical implications for strategies aimed at reducing AFB1 toxicity. Since CYP-mediated bioactivation is the critical step leading to the formation of the reactive AFB1-8,9-epoxide, interventions that reduce bioactivation or enhance detoxification may help mitigate toxic effects. Potential approaches include modulation of CYP activity, enhancement of glutathione-dependent detoxification pathways, and use of protective compounds such as quercetin. (Mary et al., 2012) [36].

Going forward, this approach could be improved by including molecular dynamics simulations, using better scoring methods, and combining these results with transcriptomic or proteomic data to get a more complete picture of metabolic capacity. From the RT-qPCR data, we detected expression of CYP3A5, CYP3A7 and CYP3A4 but not CYP1A2. This underscores the importance of these CYP3A isoforms in AFB1 activation but does not preclude that CYP1A2 activity is important at low AFB1 exposure levels (Guengerich et al., 2002, Gallagher et al., 1996) [16,28]. However, we do not know which additional detoxification enzymes, such as glutathione S-transferases, interact with these CYPs to modulate AFB1 adduct formation in VERO-E6 cells, nor the full range of AFB1 metabolites that are generated that may contribute to toxicity. Future studies should include direct functional validation of CYP activity using isoform-specific probe substrates, selective chemical inhibition, or recombinant enzyme assays to confirm the contribution of individual CYP isoforms to AFB1 bioactivation. In addition, metabolite profiling and targeted analysis of AFB1 epoxide-derived products would further clarify the enzymatic pathways driving DNA adduct formation and toxicity in VERO-E6 cells. A limitation of this study is that VERO-E6 cells are kidney-derived non-hepatic cells and therefore do not fully recapitulate hepatic AFB1 metabolism. Consequently, the metabolic activity observed in this model should be interpreted as reflecting non-hepatic bioactivation potential rather than liver-specific metabolism.

## 4. Conclusions

This study demonstrates that VERO-E6 cells support AFB1 bioactivation, as evidenced by CYP gene expression, AFB1-induced cytotoxicity, and LC-MS/MS detection of AFB1-derived DNA adducts. AI-based structural modeling and molecular docking suggest that CYP3A family enzymes, particularly CYP3A4, may contribute to this process. However, these computational approaches cannot establish catalytic activity or identify the specific CYP isoform responsible for AFB1 activation. Future studies should focus on functional validation of specific CYP isoforms and extension of this approach to other toxicants and pharmaceuticals.

## 5. Materials and Methods

### 5.1. Chemicals and Media

AFB1 was purchased from Sigma and dissolved in dimethyl sulfoxide (DMSO) at a concentration of 10 mg/mL and stored at 4 °C. The Dulbecco’s Modified Eagle Medium (DMEM) medium was purchased from CellTreat. Fetal bovine serum (FBS) was purchased from MidSci.

### 5.2. Cell Lines and Toxicity Assays

VERO-E6 cells were purchased from the American Type Culture Collection (ATCC).

VERO-E6 (ATCC CRL-1586; VERO C1008) is a clonal derivative of the VERO 76 cell line. Different VERO sublines exhibit distinct biological properties, and VERO-E6 is widely used due to its stable monolayer growth and high susceptibility to viral infection, particularly in SARS-CoV-2 studies. Cells were cultured in DMEM high glucose, 5% fetal bovine serum, and penicillin/streptomycin and incubated at 37 °C, 5% CO_2_. To assess the toxicity in VERO E6 cells with different toxicants, 50 000 cells were seeded per well in a 6-well plate and allowed to attach overnight. Cells were then treated with AFB1 (0.1% DMSO, 5 nmol/L, 100 nmol/L, and 200 nmol/L) for 6 days, 10 days, or 14 days with continuous exposure throughout the treatment period. Media were changed every third day, and fresh AFB1 at the indicated concentrations was re-added with each media change. After exposure, cells were rinsed with PBS, trypsinized, and counted using a hemocytometer to determine the total cell count. Cell lethality was measured using trypan blue (0.4%, 1:1), a dye that stains non-viable cells. Each experiment was conducted in triplicate to ensure consistent results. To evaluate the effects of AFB1 concentration and exposure time on cell proliferation and viability, statistical analysis was performed using two-way ANOVA. When significant effects were detected, Dunnett’s multiple comparison test was used to compare each AFB1-treated group with the corresponding DMSO control at each time point. Data are presented as mean ± SD from three independent biological replicates (*n* = 3), and *p* < 0.05 was considered statistically significant (Motulsky et al., 2018) [37].

### 5.3. Quantitative RT-PCR

Total RNA was isolated from cells using Trizol reagent (1 mL per 5 × 10^6^ cells) following standard procedures (Chomczynski and Mackey, 1995) [38]. Briefly, phase separation was conducted using chloroform, followed by centrifugation at 11,500 rpm for 15 min at 4 °C. The aqueous phase was collected, and RNA was precipitated using 0.5 mL of isopropanol. The resulting pellet was washed with 75% ethanol, air-dried, and then resuspended in nuclease-free water. To remove any remaining genomic DNA, the samples were treated with DNase I (Thermo Scientific, New York, NY, USA, Lot# 2893971).

Quantitative RT-PCR was then conducted using the Power SYBR Green RNA-to-CT One-Step Kit (Applied Biosystems, Waltham, MA, USA, Lot# 1707101) on a Step OnePlus Real-Time PCR System (Applied Biosystems). Each reaction contained 300 ng RNA in a final volume of 20 µL and was performed in 96-well plates according to the manufacturer’s instructions. The cycling program consisted of reverse transcription at 48 °C for 30 min, an initial denaturation at 95 °C for 10 min, followed by 50 cycles of 95 °C for 15 s, 56 °C for 15 s, and 60 °C for 1 min. A final extension was performed at 60 °C for 10 min.

During amplification, SYBR Green fluorescence was measured at each PCR cycle using the StepOnePlus Real-Time PCR System. The cycle threshold (Ct) was defined as the PCR cycle number at which the fluorescence signal from the amplified product crossed a fixed threshold above background fluorescence. Ct values were automatically calculated by the instrument software using the same threshold settings across samples. Lower Ct values indicate higher starting mRNA abundance because fewer amplification cycles are required for the fluorescence signal to reach the threshold. GAPDH was used as the housekeeping reference gene for the comparison of relative transcript abundance, as it showed stable expression with minimal variation across experimental conditions.

cDNA amplification was performed using gene-specific primers. The following forward (F) and reverse (R) primers were used: CYP3A4 (F: 5′-AAACAAAAGCACCGAGTGGA-3′; R: 5′-GGAGAGTGGTGCTAGTTGTG-3′), CYP3A7 (F: 5′-ACTGTCTACAGCCTGTGCCTGG-3′; R: 5′-TGAGCTCTAGAGAGGGCACTT-3′), CYP1A2 (F: 5′GCACAACAAGGGACACAACA-3′; R: 5′-TCCAGTTGCTGTAGCAGGATG-3′), CYP1B1 (F:5′ATCCCAATTCAAGCGCTCCT-3′; R: 5′-TGGTGCTCATGCTGCGG-3′), CYP3A5 (F: 5′-CAGCCTGGTGCTCCTCTATC-3′; R: 5′-ACCATCTTGCGTTCTCCACAT-3′). The resulting cDNA products are in the range of 150 bp to 200 bp for glyceraldehyde-3-phosphate dehydrogenase (GAPDH), CYP3A5, CYP1B1, CYP3A7, and CYP3A4 and 270 bp for CYP1A2.

Statistical analysis of RT-qPCR data was performed using ΔCt values, calculated as Ct_target − Ct_GAPDH. Relative transcript abundance was calculated using the 2^−ΔCt^ method for data visualization. Statistical comparisons among CYP isoforms were performed using one-way analysis of variance (ANOVA) followed by Tukey’s multiple comparison test. ΔCt values were used for statistical analysis because they are normalized and more appropriate for parametric comparisons than raw Ct values. Results are presented as mean ± standard deviation (SD) from three independent technical replicates (*n* = 3), and *p* < 0.05 was considered statistically significant.

### 5.4. Detection and Quantification of Aflatoxin B1 (AFB1)-Derived DNA Adducts

The metabolically active form of AFB1, the AFB1 exo-8,9-epoxide, reacts with the N^7^ group of guanine to form the AFB1 N^7^-Gua adducts (Figure 1). The AFB1 N^7^-Gua adduct is highly unstable and converts to trans-AFB1-FAPY-Gua adducts, with the combined levels representing the total DNA adducts (Dohnal et al., 2014) [22]. To assess metabolic activation of AFB1, we quantified total AFB1-associated DNA adducts in exposed cells. Approximately 2 × 10^7^ VERO-E6 cells were cultured in T75 flasks and exposed to 200 nmol/L AFB1 for 2 h, 4 h, or 16 h. After treatment, cells were rinsed twice with phosphate-buffered saline (PBS), detached using trypsin, and washed again with PBS. Genomic DNA was purified using a magnetic bead-based extraction procedure according to the manufacturer’s recommendations with Gen Find V3 mammalian DNA extraction kit (Beckman Coulter Life Science, Indianapolis, IN, USA). DNA was precipitated with 0.1 mol/L ammonium acetate in 70% ethanol and resuspended in ethanol for concentration determination. The isolated DNA was then subjected to acid hydrolysis, and AFB1-associated adducts were quantified by liquid chromatography–tandem mass spectrometry as previously reported by Jaruga et al. (2023) [39]. The data represent replicate measurements from independent biological experiments. For time-course studies, total DNA adduct levels were analyzed using one-way analysis of variance (ANOVA) to assess the effect of time. When significance was detected (*p* < 0.05), Dunnett’s post hoc test was performed to compare each time point with the 2 h reference condition (Zar et al., 2010, Montgomery et al., 2019) [40,41].

### 5.5. Computational Modeling and Docking Methodology

Protein structures for CYP3A4, CYP3A5, and CYP3A7 from *C. sabaeus* were predicted using AlphaFold3 to enable structural analysis in the absence of experimentally resolved VERO CYP structures (Jumper et al., 2021) [23]. We used AlphaFold3 to generate structural models of the VERO CYP enzymes, which we then used for computational analysis. Without these structures, it would have been difficult to do the docking or make sense of the interactions.

These models also helped with the docking in AutoDock Vina version 1.1.2, since they gave us a better idea of what the proteins look like. As a result, we were able to obtain more consistent predictions for both binding affinity and ligand orientation within the active site. This also allowed us to look more closely at protein–ligand interactions, including identifying key residues involved in binding and checking how close the ligand is to the heme iron (Fe), which is important for catalytic activity in cytochrome P450 enzymes. Details of the individual steps, including protein preparation, ligand processing, docking setup, and interaction analysis, are described in Section 5.5.1, Section 5.5.2, Section 5.5.3, Section 5.5.4, Section 5.5.5 and Section 5.5.6.

#### 5.5.1. Protein Sequence Retrieval and Structural Prediction

We obtained the amino acid sequences of cytochrome P450 enzymes from *C. sabaeus* from the NCBI protein database. To compare them with human sequences, we ran pairwise alignments between the *C. sabaeus* and *H. sapiens* CYP3A isoforms using BLASTP from the NCBI BLAST suite (https://blast.ncbi.nlm.nih.gov/Blast.cgi (accessed on 4 March 2026)). This allowed us to look at sequence identity, similarity, and whether there were any insertions or deletions (Altschul et al., 1990) [42].

For the structural predictions, we used the default settings and selected the models with the highest confidence scores (pLDDT) for further analysis. The structures were downloaded into CIF format and then converted to PDB format using a Python (version 3.10.18) -powered molecular graphics system (PyMOL) version 3.1.6.1. After that, we looked at the structures to make sure the overall folding and key features appeared reasonable.

#### 5.5.2. Heme Transfer and Template Preparation

We retrieved the experimentally determined crystal structures of the corresponding human CYP enzymes from the Protein Data Bank (PDB) (Berman et al., 2000) [43] and used them as a reference for placing the heme group, since their active sites and iron coordination are well characterized. During preparation, we removed other ligands and solvent molecules, while keeping the heme and the key residues around it. The AlphaFold-predicted VERO CYP structures were then aligned with the human CYP structures in PyMOL using the Cα backbone atoms. This helped bring both structures into the same frame, so we could compare conserved features more easily, including the cysteine residue that coordinates the heme iron. Following alignment, the heme cofactor was transferred from the human CYP structure to the Vero CYP model. The Fe–S distance between the heme iron and coordinating cysteine was measured to confirm proper coordination geometry. The resulting heme-bound structures were saved as docking-ready models.

#### 5.5.3. Protein and Ligand Preparation for Docking

We used a structure-based docking approach to look at how Aflatoxin B1 (AFB1) interacts with CYP3A4, CYP3A5, and CYP3A7. During protein preparation, we removed most of the crystallographic water molecules, except for those close to the heme or involved in ligand interactions. The heme group was kept in place. We also fixed duplicate side chains using PyMOL and MGLTools (Rosignoli & Paiardini, 2022) [44]. After that, polar hydrogens were added, Kollman charges were assigned, and the final structures were converted to Protein Data Bank, Quantum and Torsions (PDBQT) format for docking in AutoDock Vina. The three-dimensional structure of AFB1 was obtained from the PubChem database. Hydrogen atoms were then added, and Gasteiger charges were assigned. The ligand was then converted to PDBQT format, and rotatable bonds were defined for docking in AutoDock Vina.

#### 5.5.4. Docking Site Definition

For docking, we centered the search space on the heme iron in the active site. We used a cubic grid box of 4 nm × 4 nm × 4 nm (40 Å × 40 Å × 40 Å) to ensure coverage of both the heme catalytic center and surrounding substrate recognition regions. This search space allowed sampling of multiple ligand binding orientations and was applied consistently across all CYP isoforms for comparative analysis. This grid size was chosen so that both the active site and nearby regions, such as pockets and possible access pathways, were included without making the calculations unnecessarily large. Using this setup, we could check how the ligand sits in the active site, how it interacts with surrounding residues, and whether its orientation is suitable for catalytic activity.

#### 5.5.5. Docking Protocol

Docking simulations were conducted using AutoDock Vina (Eberhardt et al., 2021) [45]. To get better sampling, we ran the docking multiple times using different random seeds. In each run, up to 20 poses were generated within an energy range of 16.73 kJ/mol (4 kcal/mol). The exhaustiveness parameter was increased to 32 (default = 8) so that the program could explore a wider range of ligand conformations and provide more consistent docking results.

#### 5.5.6. Post-Docking Structural Analysis

We looked at the docking poses in PyMOL to see how the ligand sits in the active site, which residues are around it, and how close the reactive C8–C9 atoms are to the heme iron (Rosignoli & Paiardini, 2022) [44]. The C8 and C9 atoms were marked so we could track their positions, and distances from these atoms to the heme iron were measured to determine whether the orientation could support CYP-mediated activation. We also looked at which residues were within about 0.5 nm (5 Å) of the ligand to better understand the binding environment. The distances between the C8–C9 region and the heme iron were measured and, where needed, averaged so we could compare different poses. Dashed lines were mainly used for visualization, which made it easier to see how the ligand sits relative to the catalytic center. To narrow down the most relevant poses, we considered a combination of docking scores, distance measurements, and the surrounding residues. In practice, we focused on poses that showed both a reasonable binding score and a C8–C9 distance of around 0.6 nm (6 Å) or less from the heme iron, since this distance is generally needed for epoxide formation (Vasanthanathan et al., 2009) [46].

#### 5.5.7. Western Blot

Expression of CYP3A4 in yeast and mammalian cells was determined by Western blots. Yeast cells (YB437) containing pMA91-CYP3A4 (Li et al., 2009) [47] were inoculated in synthetic complete lacking leucine (SC-LEU) medium, and yeast cells lacking pMA91-CYP3A4 (YB427) were inoculated in yeast peptone dextrose (YPD) medium (Fasullo et al., 2017) [48]. Log-phase cells (A_600_ = 0.5–1) were lysed by agitation with glass beads, and protein extracts were concentrated by trichloroacetic acid (TCA) precipitation, as previously described by Foiani et al. (1994) [49]. VERO cells were washed 1X with PBS, centrifuged to collect the cell pellet, and resuspended in 500 µL of lysis buffer containing 1% Triton X-100, 0.1% SDS, 0.05 mol/L (50 mM) Tris (pH 8), and 0.001 mol/L (1 mM) EDTA. The cell suspension was sonicated for 10s, then incubated on ice for 30 min. Soluble proteins were recovered by centrifugation at 12,000 rpm for 10 min, and the protein concentration was determined using the Bradford assay. Proteins were separated on 10% acrylamide/0.266% bis-acrylamide gels and transferred to nitrocellulose membranes. Human CYP3A4 was detected by Western blots using a 1:1000 diluted mouse monoclonal anti-CYP3A4 (Invitrogen LSC169171) and a 1:10,000 diluted secondary goat anti-mouse antibody conjugated with horseradish peroxidase (Santa Cruz Biotechnology: 10 sc-2005). The signal was detected by chemiluminescence using ThermoFischer SuperSignal™ West Femto Maximum Sensitivity Substrate. Precision Plus Protein Western C Blotting Standards (Bio-Rad) were used for molecular weight standards.

## Figures and Tables

**Table 1:** Comparison of *H. sapiens* and *C. sabaeus* CYP3A4, CYP3A5, and CYP3A7 protein structures.

CYP	Overall Fold	Catalytic Core	B–C Loop	F–G Loop	Active-Site Pocket
CYP3A5 *H. sapiens*	Canonical P450 fold	Conserved	Compact	Constrained	Defined
*C. sabaeus*	Canonical P450 fold	Conserved	Slight flexibility/shift	Extended/flexible	Slightly open
CYP3A4 *H. sapiens*	Canonical P450 fold	Conserved	Compact	Constrained	Defined
*C. sabaeus*	Canonical P450 fold	Conserved	Loop extension/relaxed entrance	Extended access-channel region	Slightly open
CYP3A7 *H. sapiens*	Canonical P450 fold	Conserved	Compact	Constrained	Defined
*C. sabaeus*	Canonical P450 fold	Conserved	Relaxed	Extended access-channel region	Slightly open

**Table 2:** Binding affinities of AFB1 with *Chlorocebus sabaeus* CYP3A enzymes across multiple docking modes.

Binding Affinities (kJ/mol)			
Binding Modes	CYP3A4	CYP3A5	CYP3A7
1	−68.2 (−16.3 kcal/mol)	−36.4 (−8.7 kcal/mol)	−35.1 (−8.4 kcal/mol)
2	−67.3 (−16.1 kcal/mol)	−36.4 (−8.7 kcal/mol)	−34.7 (−8.3 kcal/mol)
3	−66.1 (−15.8 kcal/mol)	−35.6 (−8.5 kcal/mol)	−34.3 (−8.2 kcal/mol)
4	−64.0 (−15.3 kcal/mol)	−34.7 (−8.3 kcal/mol)	−33.9 (−8.1 kcal/mol)
5	−63.6 (−15.2 kcal/mol)	−34.3 (−8.2 kcal/mol)	−33.5 (−8.0 kcal/mol)
6	−63.6 (−15.2 kcal/mol)	−33.1 (−7.9 kcal/mol)	−33.1 (−7.9 kcal/mol)
7	−63.6 (−15.2 kcal/mol)	−33.1 (−7.9 kcal/mol)	−33.1 (−7.9 kcal/mol)
8	−63.2 (−15.1 kcal/mol)	−33.1 (−7.9 kcal/mol)	−32.6 (−7.8 kcal/mol)
9	−62.8 (−15.0 kcal/mol)	−33.1 (−7.9 kcal/mol)	−32.6 (−7.8 kcal/mol)
10	−61.5 (−14.7 kcal/mol)	−33.1 (−7.9 kcal/mol)	−32.6 (−7.8 kcal/mol)

**Table 3:** Mode-specific residues and catalytic relevance of AFB1 binding in *C. sabaeus* CYP3A4, CYP3A5, and CYP3A7.

CYP	Binding Modes (Table 1)	ΔG (kJ/mol)	Fe–C8/Fe–C9 (nm)	Key Interacting Residues	Catalytic Interpretation
CYP3A4	1	−68.2 (–16.3 kcal/mol)	0.386/0.457 (3.86/4.57 Å)	Phe108, Phe213, Phe215, Phe241, Phe304, Leu482, Arg105, Arg212,	The best catalytic pose is supported by a conserved aromatic clamp and compact hydrophobic wall, with Phe, Arg, and Leu residues positioning AFB1 optimally near the heme for epoxidation.
CYP3A4	2	−67.3 (–16.1 kcal/mol)	1.171/1.236 (11.71/12.36 Å)	Arg105, Arg106, Phe108	High affinity with high heme distance; may stabilize binding without supporting catalysis.
CYP3A4	5	−63.6 (–15.2 kcal/mol)	0.554/0.669 (5.54/6.69 Å)	Ala370, Arg212	Borderline productive; partial steering with one distance near the upper catalytic limit.
CYP3A4	10	−61.5 (−14.7 kcal/mol)	1.416/1.296 (14.16/12.96 Å)	Asn312, Arg372, Met371, Phe57	Peripheral binding site; non-productive despite favorable interactions.
CYP3A5	1	−36.4 (−8.7 kcal/mol)	0.349/0.475 (3.49/4.75 Å)	Thr309	Productive epoxidation requires optimal heme proximity and polar steering.
CYP3A5	2	−36.4 (−8.7 kcal/mol)	0.952/0.825 (9.25/8.25 Å)	Arg105, Arg372, Glu374, Leu373	Same affinity as Mode 1 but non-productive geometry; illustrates ΔG alone is insufficient.
CYP3A5	9	−33.1 (−7.9 kcal/mol)	0.342/0.368 (3.42/3.68 Å)	Ala305, Arg105, Thr309, Phe213	Likely productive; Arg105 provides steering with optimal distances.
CYP3A5	10	−33.1 (−7.9 kcal/mol)	0.658/0.766 (6.58/7.66 Å)	Phe304, Ser119	Mostly non-productive; distances drift beyond the optimal catalytic window.
CYP3A7	1	−35.1 (−8.4 kcal/mol)	0.487/0.496 (4.87/4.96 Å)	Ala370, Leu482, Phe304, Arg372	Productive-capable; hydrophobic contacts support binding near heme.
CYP3A7	2	−34.7 (−8.3 kcal/mol)	0.323/0.441 (3.23/4.41 Å)	Ala305, Arg105	Best CYP3A7 pose; strong heme proximity with steering interaction.
CYP3A7	3	−34.3 (−8.2 kcal/mol)	1.125/1.055 (11.25/10.55 Å)	Glu374	Non-catalytic; ligand stabilized far from heme.
CYP3A7	4	−33.9 (−8.1 kcal/mol)	0.537/0.652 (5.37/6.52 Å)	Phe304	Aromatic contact but reduced catalytic reliability.

## Data Availability

The original contributions presented in this study are included in the article/Supplementary Material. Further inquiries can be directed to the corresponding author.

## Supplementary Materials

The following supporting information can be downloaded at: https://www.mdpi.com/article/10.3390/toxins18080339/s1, Figure S1: Western Blot analysis confirming CYP3A4 protein expression in VERO-E6 cells. Left panel: immunoblot for CYP3A4 (~50 kDa). Right panel: immunoblot for GAPDH (~37 kDa) as loading control. Lane A: yeast control strain (YB226); Lane B: VERO E6 cells; Lane C: YB226 expressing CYP3A4 (positive control). CYP3A4 protein was detected in VERO E6 cells and in positive control, supporting protein level expression of CYP3A4 in VERO E6 cells. Figure S2: Pairwise BLAST alignment comparing the amino-acid sequences of CYP3A4, CYP3A7 and CYP3A5 from *Homo sapiens* (subject) and *Chlorocebus sabaeus* (VERO; query). The alignment shows a high degree of sequence conservation across the full-length protein, with 93 % sequence identity and 97 % similarity in CYP3A4, 91 % sequence identity and 95 % similarity in CYP3A7 and CYP3A5. Key conserved structural motifs characteristic of cytochrome P450 enzymes are highlighted: the heme-binding motif (FGxGPRNCIG) is shown in red, the PERF motif involved in stabilizing the P450 catalytic core is indicated in green, and the EXXR motif located in the K-helix region is highlighted in blue. The conservation of these motifs demonstrates preservation of the catalytic architecture and heme-coordination environment between human and VERO CYP3A4 enzymes; Table S1: Comparative analysis of the three most catalytically relevant AFB1 docking poses in Human and VERO CYP3A isoforms.

## Abbreviations

AFB1	Aflatoxin B1
CYP	Cytochrome P450
AI	Artificial Intelligence
ANOVA	Analysis of Variance
ATCC	American Type Culture Collection
BE	Binding Energy
BLAST	Basic Local Alignment Search Tool
BLASTP	Protein Basic Local Alignment Search Tool
Ct/CT	Cycle Threshold
DMSO	Dimethyl Sulfoxide
ERK1/2	Extracellular Signal-Regulated Kinase ½
GAPDH	Glyceraldehyde-3-Phosphate Dehydrogenase
HAAs	Heterocyclic Aromatic Amines
LC–MS/MS	Liquid Chromatography–Tandem Mass Spectrometry
OTA	Ochratoxin A
PCR	Polymerase Chain Reaction
qPCR	Quantitative Polymerase Chain Reaction
ROS	Reactive Oxygen Species
SRS	Substrate Recognition Site
mRNA	Messenger RNA
