# Supplementary material for: Toxicity and DNA Adduct Formation Reinforce AI-Guided Prediction of Aflatoxin B1 Bioactivation in VERO E6 Cells
Source: Toxins (Basel). 2026 Aug 4;18(8):339. doi: 10.3390/toxins18080339 (PMC13517767; doi:10.3390/toxins18080339)
Supplement: Supplementary file 1 [file toxins-18-00339-s001.zip › toxins-4398849-supplementary.pdf]

Supplementary Materials: Toxicity and DNA Adduct Formation Reinforce AI-Guided Prediction of Aflatoxin B1 Bioactivation in VERO E6 Cells

Bharti Sangwan, Ugochukwu Okoro, Isabella Atteck, Pawel Jaruga, Chinwe Ekenna and Michael Fasullo

Table S1. Comparative analysis of the three most catalytically relevant AFB1 docking poses in human and VERO CYP3A isoforms

CYP Isoform	Parameter	Human	VERO	Comparison / Interpretation
CYP3A4	Binding modes	4, 10, 14	1, 5, 13	Different top-ranked poses with similar catalytically relevant orientations.
	Docking score (KJ/mol)	−36.0, −35.1, −34.7<br><br>(−8.6, −8.4, −8.3 kcal/mol)	−68.2, −63.6, −60.7<br><br>(−16.3, −15.2, −14.5 kcal/mol)	VERO shows substantially stronger predicted binding.
	Fe–C8 / Fe–C9 (nM)	0.401/0.463; 0.439/0.451; 0.549/0.699<br><br>(4.01/4.63; 4.39/4.51; 5.49/6.99 Å)	0.386/0.457; 0.554/0.669; 0.802/0.895<br><br>(3.86/4.57; 5.54/6.69; 8.02/8.95 Å)	Best poses position the C8–C9 bond near the heme iron in both enzymes.
	Key interacting residues	Ala305, Ile120, Ile301, Phe108, Phe215, Phe304, Ser119, Arg105, Arg372	Ala305, Arg105, Arg212, Glu374, Ile120, Ile301, Phe108, Phe215, Phe241, Phe304, Ser119	Catalytic core conserved; VERO includes additional Arg212 and Phe241.
CYP3A5	Binding modes	2, 4, 16	7, 9, 20	Different preferred poses with similar

				catalytic orientation.
	Docking score (kJ/mol)	−35.6, −34.3, −31.4<br><br>(−8.5, −8.2, −7.5 kcal/mol)	−33.1, −33.1, −31.0<br><br>(−7.9, −7.9, −7.4 kcal/mol)	Overall similar predicted binding affinity.
	Fe–C8 / Fe–C9 (nM)	0.452/0.460; 0.477/0.456; 0.408/0.498<br><br>(4.52/4.60; 4.77/4.56; 4.08/4.98 Å)	0.316/0.407; 0.342/0.368; 0.341/0.442<br><br>(3.16/4.07; 3.42/3.68; 3.41/4.42 Å)	VERO places AFB1 slightly closer to the heme iron.
	Key interacting residues	Ala305, Arg105, Arg106, Glu374, Ile301, Leu120, Phe304, Ser119, Thr309	Ala305, Ala370, Arg105, Arg106, Arg372, Glu374, Ile120, Ile301, Leu108, Leu373, Leu482, Phe304, Ser119, Thr309	Conserved catalytic residues with minor hydrophobic pocket differences.
CYP3A7	Binding modes	3, 4, 16	1, 2, 11	Highly comparable catalytically relevant poses.
	Docking score (kJ/mol)	−36.4, −35.6, −33.1<br><br>(−8.7, −8.5, −7.9 kcal/mol)	35.1, −34.7, −32.6<br><br>(−8.4, −8.3, −7.8 kcal/mol)	Nearly identical predicted binding affinity.
	Fe–C8 / Fe–C9 (nM)	0.462/0.449; 0.350/0.466; 0.365/0.487<br><br>(4.62/4.49; 3.50/4.66; 3.65/4.87 Å)	0.487/0.496; 0.323/0.441; 0.426/0.543<br><br>4.87/4.96; 3.23/4.41; 4.26/5.43 Å)	Very similar catalytic geometry.
	Key interacting residues	Ala305, Ala370, Arg105, Arg106, Arg372, Glu374, Ile120, Leu373, Leu482, Phe108, Phe304, Ser119, Thr309	Ala305, Ala370, Arg105, Arg106, Arg120, Arg372, Glu374, Gly481, Leu108, Leu482, Phe213, Phe304, Thr309	Highest structural conservation with only minor residue differences.

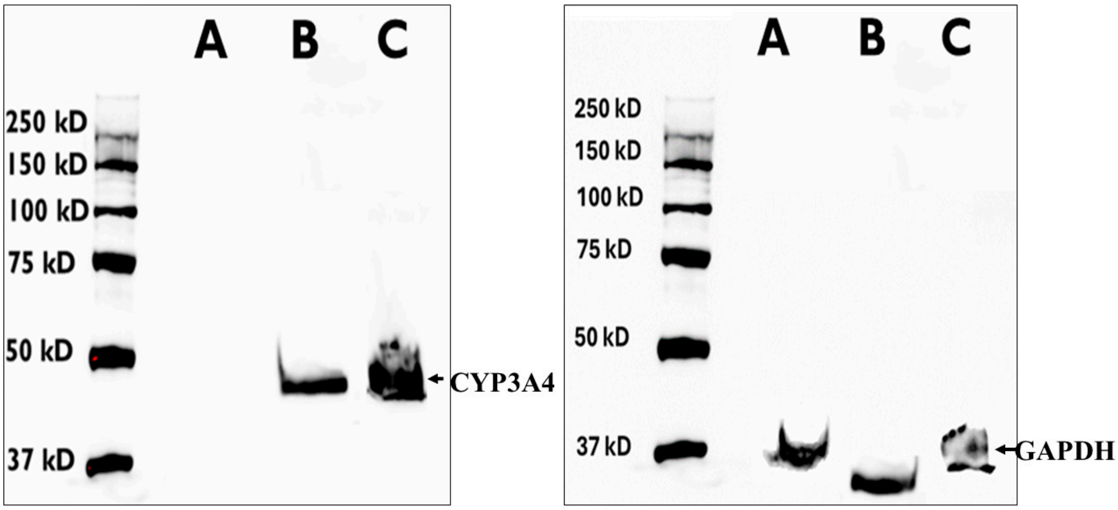

Figure S1. Western blot analysis confirming CYP3A4 protein expression in VERO-E6 cells. Left panel: immunoblot for detecting CYP3A4 (~50 kDa). Right panel: immunoblot for detecting GAPDH (~37 kDa) as loading control. Molecular weight standards are in the left lane of each immunoblot. 50 ug was loaded onto each lane. Lane A: Yeast control strain (YB226); Lane B: VERO-E6; Lane C: Yeast strain containing pMA91-CYP3A4 (YB427, positive control). CYP3A4 protein was detected in VERO-E6 cells and in the positive control, supporting protein-level expression of CYP3A4 in VERO-E6 cells.

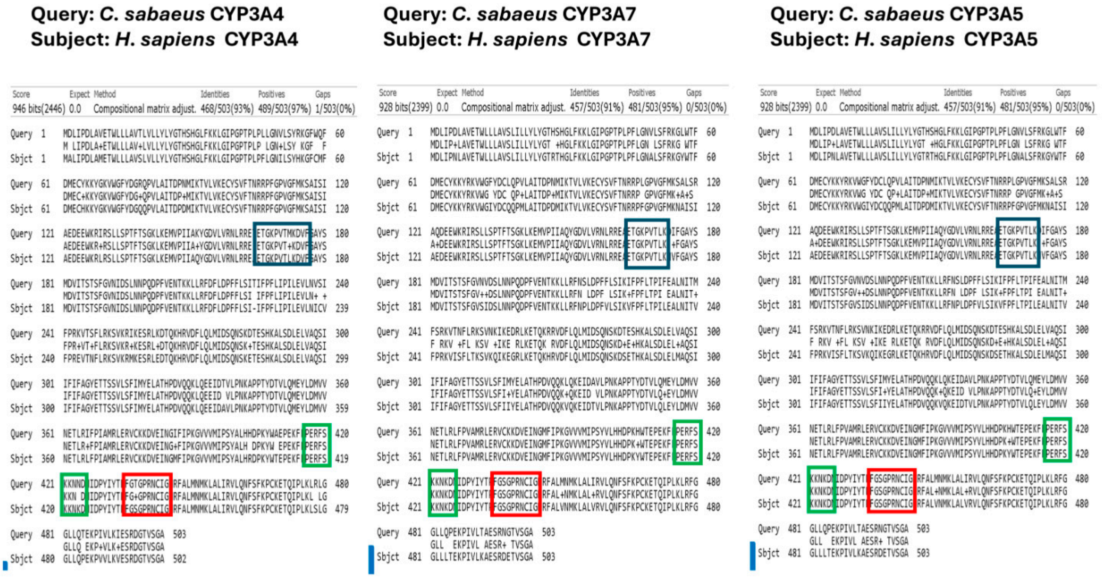

Figure S2. Pairwise BLAST alignment comparing the amino-acid sequences of CYP3A4, CYP3A7 and CYP3A5 from Homo sapiens (subject) and Chlorocebus sabaeus (VERO; query). The alignment shows a high degree of sequence conservation across the full-length protein, with 93 % sequence identity and 97 % similarity in CYP3A4, 91 % sequence identity and 95 % similarity in CYP3A7 and CYP3A5. Key conserved structural motifs characteristic of cytochrome P450 enzymes are highlighted: the heme-binding motif (FGxGPRNCIG) is shown in red, the PERF motif involved in stabilizing the P450 catalytic core is indicated in green, and the EXXR motif located in the K-helix region is highlighted in blue. The conservation of these motifs demonstrates preservation of the catalytic architecture and heme-coordination environment between human and VERO CYP3A4 enzymes.
